# Biosafety, histological alterations and residue depletion of feed administered anti-parasitic drug emamectin benzoate in golden mahseer, *Tor putitora* (Hamilton, 1822) as a model candidate fish for sport fishery and conservation in temperate waters

**DOI:** 10.3389/fphar.2023.1106124

**Published:** 2023-02-10

**Authors:** Sumanta Kumar Mallik, Shivam Singh, Neetu Shahi, Prasanna Kumar Patil, Krishna Kala, Richa Pathak, Abhay Kumar Giri, Partha Das, Ritesh Shantilal Tandel, Suresh Chandra, Nanitha Krishna, Rajisha Ravindran, Pramod Kumar Pandey

**Affiliations:** ^1^ ICAR-Directorate of Coldwater Fisheries Research (ICAR-DCFR), Bhimtal, India; ^2^ ICAR-Central Institute of Brackishwater Aquaculture (ICAR-CIBA), Chennai, India; ^3^ ICAR-Central Institute of Fisheries Technology (ICAR-CIFT), Kochi, India

**Keywords:** emamectin benzoate, golden mahseer, toxicity, anti-parasitic drug, residue depletion, withdrawal period

## Abstract

In the present experiment, the attempt has been made to study the biosafety, toxicity, residue depletion and drug tolerance of graded doses of emamectin benzoate (EB) in juveniles of golden mahseer, *Tor putitora* as a model candidate fish for sport fishery and conservation in temperate waters through an extended medicated feeding. The graded doses of EB *viz.*, 1× (50 μg/kg fish/day), 2 × (100 μg/kg fish/day), 5 × (250 μg/kg fish/day) and 10 × (500 μg/kg fish/day) were administered to golden mahseer juveniles through medicated diet for 21 days at water temperature of 18.6°C. The higher doses of EB did not cause any mortality during and 30 days after the end of medication period, but considerable variations in feeding and behavior were observed. Severe histological alterations observed after EB-diets (5 × and 10×) were vacuolation, pyknotic nuclei, melanomacrophage centre and necrosis in liver; Bowman’s capsule dilation, degenerated renal tubules in kidney; myofibril disintegration, muscle oedema, splitting of muscle fibres, migration of inflammatory cells in muscle; and abundant goblet cells, dilated lamina propria and disarrangement of mucosa in intestine tissues. The residual concentrations of EB metabolites Emamectin B_1a_ and B_1b_ were analyzed using muscle extracts and were found to be peaked during medication period followed by gradual depletion in post-medication period. The outcome of this study showed that the Emamectin B_1a_ residual concentration in fish muscle in 1×, 2×, 5×, and 10× EB treatment groups were 1.41 ± 0.49, 1.2 ± 0.7, 9.7 ± 3.3, and 37.4 ± 8.2 μg/kg at 30 days of post-medication period, respectively, which falls under the maximum residue limits (MRLs) of 100 μg/kg. The results support the biosafety of EB at recommended dose of 50 μg/kg fish/day for 7 days. As residue of EB is recorded falling within the MRL, no withdrawal period is recommended for golden mahseer.

## Introduction

The study of parasitic diseases and their controlling agent is vital for the understanding of effectiveness and usage of the drug to fish. A parasite not only affects the fish organs, but also, it can alter the behavior of host and become resistant to drugs that contribute to its survival within the host (Zander, 2013). The increased prevalence of parasitic disease in aquaculture is exacerbated by greater globalization of the trade in aquatic animals and their products (Bondad-Reantaso et al., 2005) and the other risks of translocation and establishment of parasite into new areas. The primary infection of fish ectoparasites (Argulus spp., sea lice, *Ichthyophtherius* sp) may further open new doors for the secondary bacterial or viral infections, which can be more lethal to affect fish populations (Tully and Nolan, 2002). These parasitic diseases have caused tremendous economic loss in European aquaculture including fish population of both farmed and wild region. There was a loss due to parasitic infection of about US$ 480 million per year in the world salmon industry in North America (Shinn et al., 2015) and about US$ 615.0 per hectare for 1 year in carp farms in India (Sahoo et al., 2013). The economic loss assessment data showed that reduced growth rate, carp mortality and drug expense for controlling argulosis contributed to 8%–10% of total economic loss in Indian carp farming.

**FIGURE 1:**
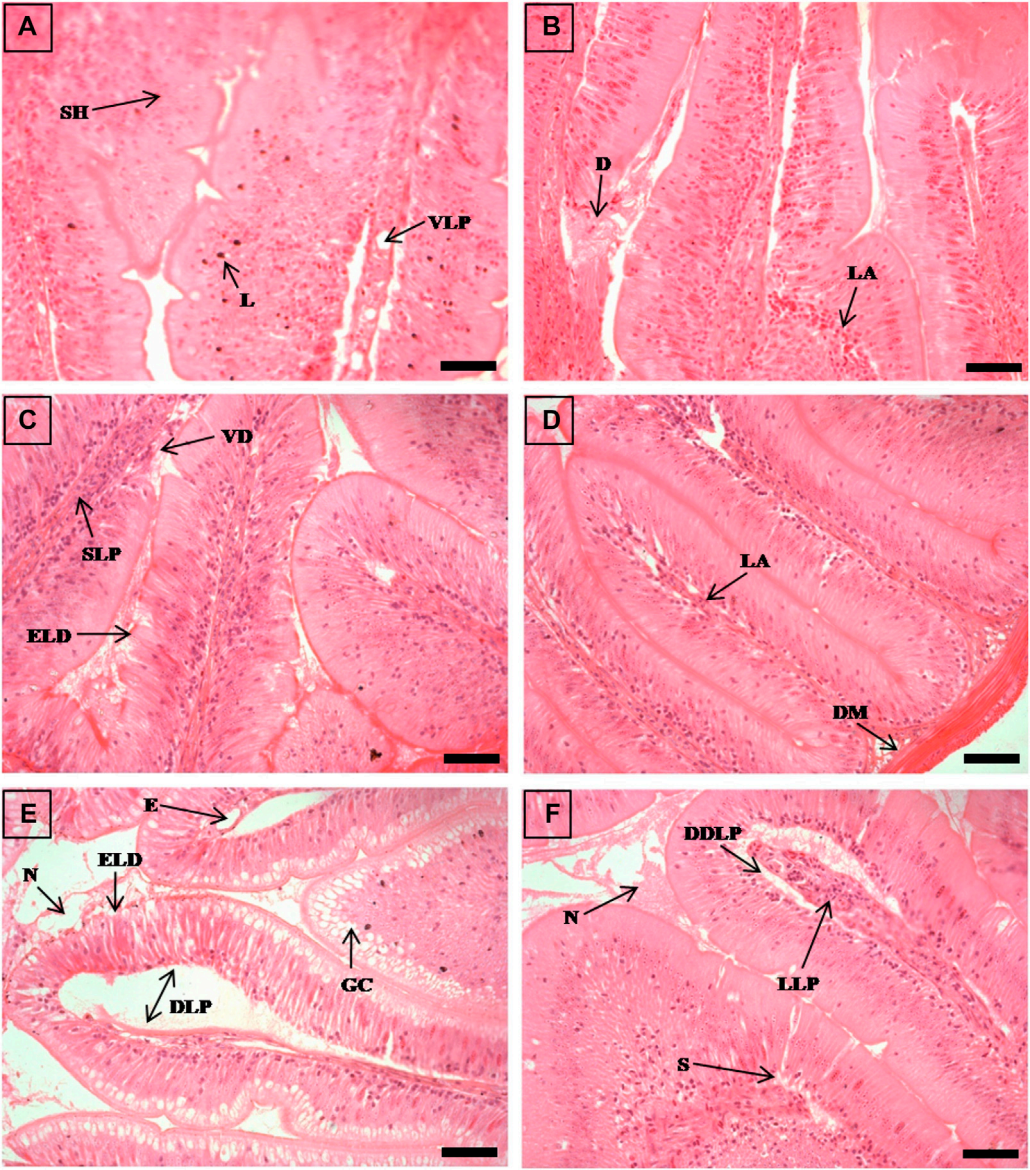
The histological sections (4 µm) of intestine tissues from EB-administered golden mahseer on 11th day **(A, B)** and 21st day **(C–F)** of EB-medication. The histological changes in intestine tissues of fishes fed with EB doses **(A)** at 250 μg/kg fish/day showing vacuolated lamina propria (VLP), submucosal hyperplasia (SH), deeply stained lymphocytes (L); **(B)** at 500 μg/kg fish/day showing degenerative changes in lamina propria and submucosa **(D)**, lymphocytes abundance (LA); **(C)** at 50 μg/kg fish/day showing swelling of lamina propria (SLP), epithelial layer disintegration (ELD), vili degeneration (VD); **(D)** at 100 μg/kg fish/day showing disarrangement of muscularis mucosa (DM), abundant lymphocytes in lamina propria (LA); **(E)** at 250 μg/kg fish/day showing abundant goblet cells (GC), extensively dilated lamina propria (DLP), epithelial layer disintegration (ELD), oedema **(E)**, necrotic debries in lumen (N); **(F)** at 500 μg/kg fish/day showing dilated and distended lamina propria (DDLP), splitting of submucosal layer (S), lymphocytes aggregation in lamina propria (LLP), necrotic debries in lumen (N). The H&E stained sections are visualized at magnification of ×400 under inverted light microscope (Olympus IX53, Canada). The scale bar value depicted in the right bottom corner **(A–F)** is 20 µm.

To control parasitic diseases such as fish louse (*Argulus* spp.), gill flukes (*Dactylogyrus* sp.), *Myxobolllus* sp., ich (*Ichthyophtherius* sp.) and gill maggot (*Ergasillus* sp.), a wide variety of drugs are available in the market and are frequently used by fish farmers. But there are very less approved drugs for parasitic diseases in many countries including India. Due to lack of licensed antiparasitic drugs and their unregulated use in aquaculture has led to the situation of drug resistant parasitic populations (Shinn and Bron, 2012). As per the record of globally used chemotherapeutic agents, which are being used in salmon farming, the eleven active ingredients can be divided into five types of pesticides, which include two urea derivatives (benzoylphenylureas): teflubenzuron and diflubenzuron; two organophosphates: dichlorvos and azamethiphos; three avermectins: ivermectin, emamectin benzoate and doramectin; an oxidizing agent: oxygen peroxide and three pyrethrine: pyrethrum, cypermethrin and deltamethrin (Roth, 2000). In India, EB, Nuvan, Butox Vet, Cliner, Ectodel (2.8%), Hitek Powder and Paracure-IV are commonly used drugs to control parasitic diseases in aquaculture (Mishra et al., 2017). Among different antiparasitic formulations, emamectin benzoate is considered as safe and an effective in-feed anti-parasitic agent for controlling parasitic infestations both in marine and freshwater-reared fish and has been approved by the United States Food and Drug Administration (USFDA) and European Medicines Agency (EMA). It is a semi-synthetic derivative of a chemical produced by the bacterium, *Streptomyces avermitilis*. It belongs to a class of compounds called avermectins, which are commonly used as an anti-parasitic agent against internal and external parasites in a variety of host species, particularly mammals. It consists of two chemicals with a similar structure (Supplementary Data S1: Figure 1: Chemical structure of emamectin benzoate designed by ACD/ChemSketch) mixed with minimum of 90% 4″ -epimethylamino-4″-deoxyavermectin B_1a_ and a maximum of 10% 4″-epi-methylamino-4″-deoxyaverrnectin B_1b_ benzoate (Leibee et al., 1995). The molecular formula of EB is stated in the form of Emamectin B1a: C49H75NO13 and Emamectin B1b: C48H73NO13. The mechanism of action of emamectin is similar to other available avermectins. The binding of emamectin with gamma-aminobutyric acid (GABA) receptors and glutamate-gated chloride channels leads to its blockage which leads to neuromuscular paralysis of parasites (Roberts and Hutson, 1999; CAHS, 2007). In 2000, for the first time, EB was registered as an antiparasitic veterinary drug in the United Kingdom for its application in temperate aquaculture and used as a feed additive in a product formulation under the trade name Slice (Schering-Plough Animal Health) (Singha et al., 2022). The European Medicines Evaluation Committee (EMEC) has set the maximum residue limit (MRL) of EB 100 μg/kg in muscle and fillet and that is considered safe for the human consumption. In the past few years, EB has been used as medicated feed in all jurisdictions. In fact, EB is the mostly used product in Canada (under Emergency Drug Release) and the US (Investigational New Animal Drugs-INAD) for control of parasitic infestations in fish (Chowdhury et al., 2012). Considering, the enhanced attention towards use of EB by global aquaculture practitioners, the safety study is need to be carried out in different fish species and climates in order to combat the parasite resistance towards this drug, toxicity and unregulated use in fish farms. The biosafety and residue depletion studies have been carried out in fish of different climatic region using recommended dose i.e. 50 μg/kg fish per day fed for 7 consecutive days. The toxicity of EB on rainbow trout and Atlantic salmon has been studied by various researchers in different regions and habitats (Roy et al., 2000; Stone et al., 2000; Stone et al., 2002). There are reports of EB toxicity and biosafety in common carp (Braun et al., 2008), Asian Seabass (Raja et al., 2020), *Labeo rohita* (Choudhary et al., 2022; Kumar et al., 2022) and Nile tilapia (Julinta et al., 2020) from Indian freshwater and brackishwater aquaculture system.

Golden mahseer (*Tor putitora*) is widely distributed in South and South-East Asia and regarded as an important food, game and sport fish in Afghanistan, Bangladesh, Bhutan, India, Iran, Myanmar, Nepal, Pakistan, Thailand and Sri Lanka (Islam and Tanaka 2007). The prevalence of fish ectoparasites such as *Argulus* sp. And *Trichodina* sp. Are reported in mahseer from Indonesia and India (Mallik et al., 2010; Muchlisin et al., 2014), where infection rate was found to be around 50% of the affected fish populations. Myxozoan infection by parasite *Myxobolus tambroides*, a species novel, was reported in Malaysian mahseer, *Tor tambroides* from Tasik Kenyir Reservoir, Malaysia (Székely et al., 2012). But, there is hardly any information available on application of EB for the control of parasitic infection, biosafety and tissue depletion in golden mahseer. Even, information is scanty on drug tolerance of fish to the higher concentrations of EB administration for an extended medication period of 21 days (three times the recommended duration of EB application). Considering the above information, the present experimental trial is aimed to determine the effect of EB on biosafety, toxicity, residue depletion and drug tolerance in juvenile golden mahseer as a model candidate coldwater fish species for sport fishery and conservation. The experiment is conducted through an extended medicated feeding of graded doses of EB to golden mahseer for 21 consecutive days at 1 × (50 μg/kg fish/day), 2 × (100 μg/kg fish/day), 5 × (250 μg/kg fish/day) and 10 × (500 μg/kg fish/day) concentrations. The study is also intended to improve and fills the knowledge gap on the correct usage of this drug in coldwater aquaculture system and consumption of fish meat after the drug withdrawal.

## Materials and methods

### Fish collection and acclimatization

The juveniles of golden mahseer, *Tor putitora* (average size 20.8 ± 2.4 cm and average body weight 78.6 ± 10.65 g) were brought from the mahseer breeding facility of ICAR- Directorate of Coldwater Fisheries Research (ICAR- DCFR) and acclimatized for 7 days before proceeding for EB biosafety experiment. They were acclimatized in 500 L rectangular tanks with continuous water flow rate of 1.0 L min^-1^ and monitored for any abnormality and diseases. The fish with no significant disease sign and parasite infection were shortlisted and used for EB medication. During acclimatization phase of 15 days, the fish were divided into fifteen tanks with 30 nos each and fed with 2% body-weight feed every day. To study the EB biosafety, total five experimental groups, *viz.*, negative control (EB free diet), 1 × (50 μg/kg fish/day EB-diet), 2 × (100 μg/kg fish/day EB-diet), 5 × (250 μg/kg fish/day EB-diet) and 10 × (500 μg/kg fish/day EB-diet) in triplicates were employed. In order to study the tolerance of fish to varied concentrations of EB, the drug was tested at higher level along with the recommended dose 50 μg/kg fish/day EB-diet. The tanks were subjected to water exchange and siphoning twice a day and water quality parameters such as temperature, pH, dissolved oxygen, nitrite, nitrate, orthophosphate, ammonia, alkalinity, iron, calcium hardness, oxidative reductive potential and conductivity were estimated twice a week by titration method as well as using multi-parameter waterproof meter (Hanna Instruments Inc., United States).

### EB-medicated diet administration

The medicated feed was prepared for five different treatment groups, *viz.*, negative control, 1×, 2×, 5×, and 10× doses of EB (Table 1). The EB coating on the surface of feed pellet was done by using vegetable oil (5% v/w of feed) as a binding agent followed by drying at room temperature for 10 h for efficient binding. The different doses of EB (Sigma-Aldrich, India: Cat no 31733-250 MG) and feed (2%) were calculated according to the fish body weight per day for each experimental tank (Table 1). The total feed diet per day for every experimental group was split into two equal parts and fish were fed *ad libitum* twice a day (9:00 and 17:00 h). During the medication period, control group and all treatment group fish were fed with EB-free and EB-medicated feed for 21 days, respectively. After 21 days of medicated feeding period, EB-free diet was given to all fishes (control as well as treatment groups) and monitored for further 30 days.

**TABLE 1:** Feed composition for golden mahseer juveniles (crude protein: −35%; crude lipid: −8.7% of feed).

	Feed ingredients	g per kg of diet except for EB				
		Control	1× (50 μg/kg)	2× (100 μg/kg)	5× (250 μg/kg)	10× (500 μg/kg)
1	Fish meal	300.00	300.00	300.00	300.00	300.00
2	Soyabean meal	200.00	200.00	200.00	200.00	200.00
3	Wheat flour	430.00	430.00	430.00	430.00	430.00
4	Soy oil	50.00	50.00	50.00	50.00	50.00
5	Fish oil	10.00	10.00	10.00	10.00	10.00
6	Vitamin/Mineral mix^*^	10.00	10.00	10.00	10.00	10.00
7	EB^#^	0.0 mg	2.5 mg	5.0 mg	12.5 mg	25.0 mg

^*^Composition of vitamin-mineral mix (quantity/kg mix).

Vitamin sources: Thiamin hydrochloride, 1.0 g; riboflavin, 1.5 g; pyridoxine hydrochloride, 1.0 g; cyanocobalamine, 0.002 g; nicotinic acid, 1.0 g; folic acid, 0.2 g; myo-inositol, 50.0 g; D-biotin, 0.1 g; calcium pantothenate, 2.0 g; L-ascorbyl-2-triphosphate, 50.0 g; retinal acetate, 0.075 g; cholecalciferol, 0.006 g; cellulose 393.117 g, tocopherol acetate, 0.1 g and choline chloride, 1.0 g.

Mineral sources: Calcium carbonate, 50 g; magnesium oxide, 124.0 g; CaHPO_4_.2H_2_O, 200.0 g; ferric citrate, 20.0 g; potassium iodide, 0.4 g; zinc sulphate, 4.0 g; copper sulphate, 3.00 g; manganese sulphate 3.0 g, KH_2_PO_4_, 300.0 g; cobalt sulphate, 0.2 g; sodium selenite, 0.3 g; sodium chloride, 40.0 g; CMC, 255.1 g.

^#^EB, is calculated according to treatment dose per kg of fish biomass and mixed with feed of 2% body weight per kg for each treatment groups.

### Monitoring of feeding and behavioral changes

In the entire biosafety experimental period (15 days acclimatization, 21 days of EB medication and 30 days of residue depletion study), feeding and animal behavior was recorded every day. To study the animal behavior, parameters such as gasping of air, hyperactivity, position in water column, flashing, lethargy and abnormal pigmentation was considered. The fish were also monitored for any gross pathological sign on their body surfaces. Survival percentage of control and all the treatment group fishes was recorded in EB medication and post-medication period. To study the feeding behavior of fishes, the unconsumed feed from each tank was collected separately after 1 h of feed broadcast every day, dried under hot air oven at 40–45°C for 18–20 h then measured carefully. Depending on consumption of feed, feeding behavior of fish was rated using a scale ranging from 0 to 4 as stated below.

o Approximately no feed consumed: 0
o Approximately 25% feed consumed: 1
o Approximately 50% feed consumed: 2
o Approximately 75% feed consumed: 3
o Approximately 100% feed consumed: 4

### Fish sacrifice and histopathological examination

For studying histopathological changes in EB-fed golden mahseer, the intestine, liver, kidney and muscle tissues were collected after 11th and 21st day of medication period by euthanizing the fishes with MS-222 (Tricaine methanesulfonate, HiMedia). Three fish from each experimental dose were collected and pooled separately for each tissue sample collection. The collected fish samples of intestine, liver, kidney and muscle tissues were immediately fixed in Davidson’s fixative for 18–24 h and the samples were stored in 70% ethanol until further processing. Histological procedure was followed according to the protocol (Mumford et al., 2007) with slight modifications. The 4.0 μm sections of tissue samples were prepared by using microtome (Microm HM 323, Thermo Scientific) and adhered to glass slides using egg albumin:glycerol (1:1) followed by baking at 37°C for 1 h. The slides were stained with hematoxylin and eosin and mounted using DPX (Dibutylphthalate Polystyrene Xylene). The histopathological changes were examined by visualizing under the inverted light microscope (Olympus IX53, Canada) with Camera Q-IMAGING, 01-MP 3.3- R- CLR-10, Color RTV10 BIT, Light source OLYMPUS, TH4-200.

## LC-MS/MS based residue analysis of EB administered fish tissues

### Sample extraction

The muscle tissues from EB-fed fish (3 nos. Of fish from each treatment) were collected during medication period (after day 11 and 21) and post-medication period (day 31, 41, and 51) for residue depletion analysis. The samples were homogenized and 5 g weighed samples were collected in 50 mL centrifuge tubes. Samples were prepared by fortifying blank samples with the appropriate volumes of standard solutions and kept for 5 min. After that the Samples were extracted by adding10 mL Acetonitrile with 1% acetic acid and 1 g sodium acetate and vortex mixed for 1 min. The samples were centrifuged at 4,000 rpm for 10 min at 4°C and the supernatant was collected. Total 1.5 mL supernatant was taken into 2 mL tube (150 mg MgSO_4,_ 25 mg PSA, 25 mg C_18_) and the tubes were centrifuged at 4,000 rpm for 5 min at 4°C. The supernatant was collected and filtered through 0.2 PTFE filter membrane into LC-MS vials.

### LC-MS/MS conditions

The experiment employed LC-MS/MS 4000 (Applied Biosystems/MDS Sciex, CA, United States) Triple quadrupole mass spectrometer. Water with 0.1% formic acid (A) and Acetonitrile with 0.1% formic acid (B) selected as the mobile phase. Kinetex 2.6 µm C18 100Å (100 × 2.1 mm) column was used for separation. The column temperature operated at 40°C and compound-dependent parameters were tuned for optimization of the multiple reactions monitoring method using direct infusion analysis. The injection volume was 10 µL. The standard curve was prepared for Emamectin B_1a_ and B_1b_ and their respective residue concentrations in muscle tissues were calculated.

### Optimization of instrumental parameters

Compound optimization using manual tuning mode in QTRAP LC-MS/MS was used for the development of multiple reaction monitoring parameters for EB analysis. The solvent standard prepared in methanol was infused directly in to the MS at a rate of 10 µL per minute and at a concentration of 1 µg mL^-1^ and the software ramps up various instrument parameters in order to identify optimum settings for each of the most three abundant product ions. Prominent molecular masses were tuned and identified as m/z 887.00 and 872.49 Da for Emamectin B_1a_ and B_1b_ with three most intense product ion spectra. The optimized source and gas parameters were as follows: curtain gas (CUR) 20; collision gas (CAD) medium; ion source temperature (TEM), 450°C; ion source gas 1 (GS1), 45; ion source gas 2 (GS2), 50; ion spray voltage, +4500 V and Dwell time, 100 milli seconds. Compound-dependent parameters including the de-clustering potential (DP), the collision energy (CE), the entrance potential (EP) and the collision exit potential (CXP) were stated as Appendix A. Supplementary Data. S1

### Validation of parameters: Validated recovery percentage (R %), limits of detection and limits of quantification

The recovery experiments involved spiking the blank matrix with standards. The percent recovery was calculated from the equation as stated below:

R % = [Observed concentration of spiking sample]/[Expected concentration] ×100 was used to calculate the percent recovery.

The linearity of the calibration curves was used to estimate LOD and LOQ. They were calculated using the slope and standard deviation (σ) of the analytical curve’s linear coefficient intercept as follows:

LOD: 3 × (standard deviation of intercept/slope) and LOQ = 10 × (standard deviation of intercept/slope).

### Statistical analysis

Graph preparation and calculation of significance statistical data were performed using GraphPad software (Prism, version 5.01). The error bar values in EB residue graph were calculated as standard error means (SEM) of the data. The rating of feeding behavior was expressed as mean ± standard deviation and one-way analysis of variance (ANOVA) was performed using IBM Statistical package for social science (SPSS) software version 19.0 (SPSS Inc, Chicago IL) to find significance difference (*p* < 0.05) between the graded levels of EB.

## Results

### Feed consumption post EB treatment

All the fishes showed normal feeding during acclimatization period. During the EB medication period of 21 days, the fishes treated with 1 × and 2 × doses of EB continued to intake 100% feed even after the medication period (Table 2). The fishes treated with 5 × and 10 × doses of EB for 21 days showed considerable decrease in feed intake during and after the EB medication period. Discontinuation of EB-diet after medication period and feeding with EB-free diet showed the slow increase in feed intake by 5 × and 10 × EB treated fish (Table 2). The water quality parameters during this study were in optimal range (Table 3).

**TABLE 2:** Rating of feeding behavior of golden mahseer juveniles administered with graded doses of EB (1×-10×) and control fish fed with normal feed.

Sr No.	Treatment group	Acclimatization period (15 days)	Medication period (EB diet for 21 days)		Post-medication period (EB-free diet for 30 days)		
			0–11	12–21	22–31	32–41	42–51
1	Control	4.0 ± 0.0	4.0 ± 0.0	4.0 ± 0.0	4.0 ± 0.0	4.0 ± 0.0	4.0 ± 0.0
2	1× (50 μg/kg)	4.0 ± 0.0	4.0 ± 0.0a	4.0 ± 0.0a	4.0 ± 0.0a	4.0 ± 0.0	4.0 ± 0.0
3	2× (100 μg/kg)	4.0 ± 0.0	4.0 ± 0.0a	4.0 ± 0.0a	4.0 ± 0.0a	4.0 ± 0.0	4.0 ± 0.0
4	5× (250 μg/kg)	4.0 ± 0.0	2.0 ± 0.4b	2.0 ± 0.1b	2.0 ± 0.6b	3.0 ± 0.92	4.0 ± 0.0
5	10× (500 μg/kg)	4.0 ± 0.0	2.0 ± 0.8b	1.0 ± 0.3b	1.0 ± 0.8b	3.0 ± 0.16	3.0 ± 0.87

Columns not sharing the same superscript “a” and “b” varied significantly (*p* < 0.05).

**TABLE 3:** Physico-chemical parameters of the golden mahseer tanks during the EB medication and post-medication period. All the parameters were estimated in duplicate, and during forenoon, twice a week for both control and EB treated groups.

Sr No.	Water quality parameters	Detected range
1	Dissolved Oxygen	7.7 ± 0.84 mg L^-1^
2	Calcium Hardness	60.0 ± 5.0 mg L^-1^
3	Nitrate	11.5.0 ± 0.5 mg L^-1^
4	Nitrite	11.0 ± 1.0 mg L^-1^
5	Iron	0.3 ± 0.1 mg L^-1^
6	Ammonia	0.02 ± 0.25 mg L^-1^
7	Orthophosphate	0.5 ± 0.1 mg L^-1^
8	Alkalinity	85.0 ± 5.0 mg L^-1^
9	Oxidative reductive potential	248.0 ± 3.0 Mv
10	Conductivity	150.0 ± 10.0 μs.cm^-1^
11	pH	7.5–8.0
12	Temperature	18.6°C ± 2.25°C

### Behavioral characteristics, survivability and fitness of EB treated golden mahseer

No mortalities were observed in control and EB administered fish during acclimatization, medication and post-medication period. The 10 × EB treated fish showed abnormal pigmentation on their body surfaces during the 12–21 days of medication period. Normal behavior was observed for the control, 1 × and 2 × EB administered groups. The fish treated with 5 × and 10 × EB doses showed abnormal behavior such as lethargy, gasping of air and crowding near the inlet of water at the end of medication and initial phase of post-medication period. No external lesions were observed in control and EB treated fish.

### Histopathological alterations due to high EB doses

**FIGURE 2:**
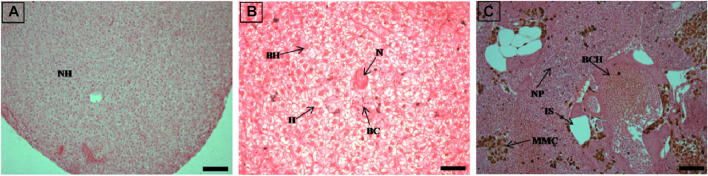
The histological sections (4 µm) of liver tissues from control fish showing normal hepatocytes **(A)** and EB-administered golden mahseer on 11th day **(B, C)** of EB-medication. The histological changes in liver tissues of fishes fed with EB doses **(B)** at 250 μg/kg fish/day showing hepatocyte hypertrophy (H), binucleated hepatocytes (BH), blood congestion (BC), necrosis (N); **(C)** at 500 μg/kg fish/day showing large melanomacrophage centres (MMC), blood congestion in hepatic veins (BCH), increased sinusoids (IS), nuclear pyknosis (NP). The H&E stained sections are visualized at magnification of 200 **(A and C)** and 400X **(B)** under inverted light microscope (Olympus IX53, Canada). The scale bar value depicted in the right bottom corner is 20 µm **(B)** and 40 µm **(A and C)**.

**FIGURE 3:**
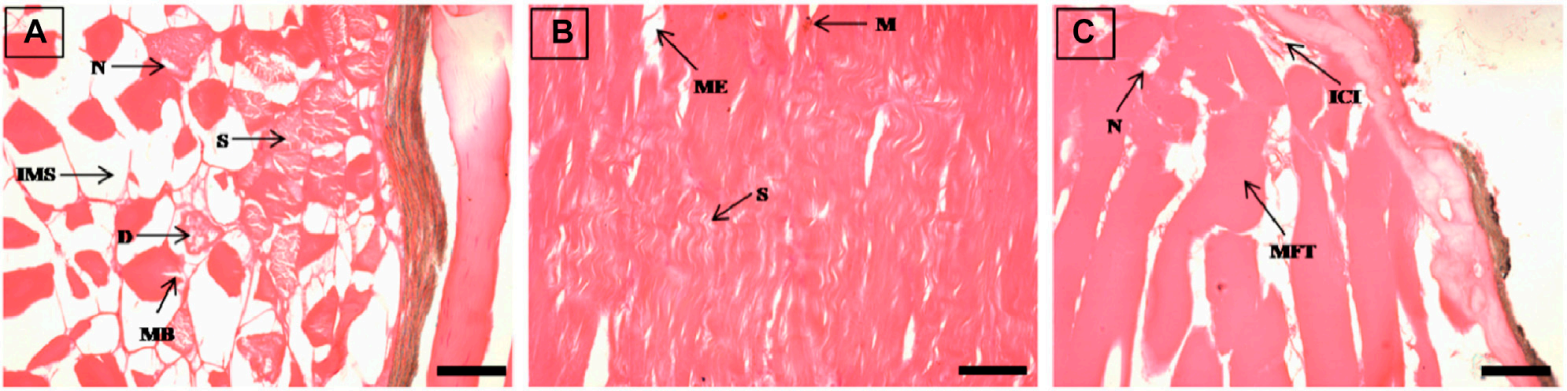
The histological sections (4 µm) of muscle tissues from EB-administered golden mahseer on 11th day **(A–C)** of EB-medication. The histological changes in muscle tissues of fishes fed with EB doses **(A)** at 100 μg/kg fish/day showing degeneration of muscle bundles (D), splitting of myofibrils (S), broken muscle bundles (MB), increased inter muscular space (IMS), necrosis (N); **(B)** at 250 μg/kg fish/day showing muscular oedema (ME), severe splitting of myofibrils (S), melanomacrophages (M); **(C)** at 500 μg/kg fish/day showing necrosis (N), muscle fibres thickening (MFT), inflammatory cells infiltration towards broken myofibrils (ICI). The H&E stained sections are visualized at magnification of ×400 under inverted light microscope (Olympus IX53, Canada). The scale bar value depicted in the right bottom corner **(A–C)** is 20 µm.

**FIGURE 4:**
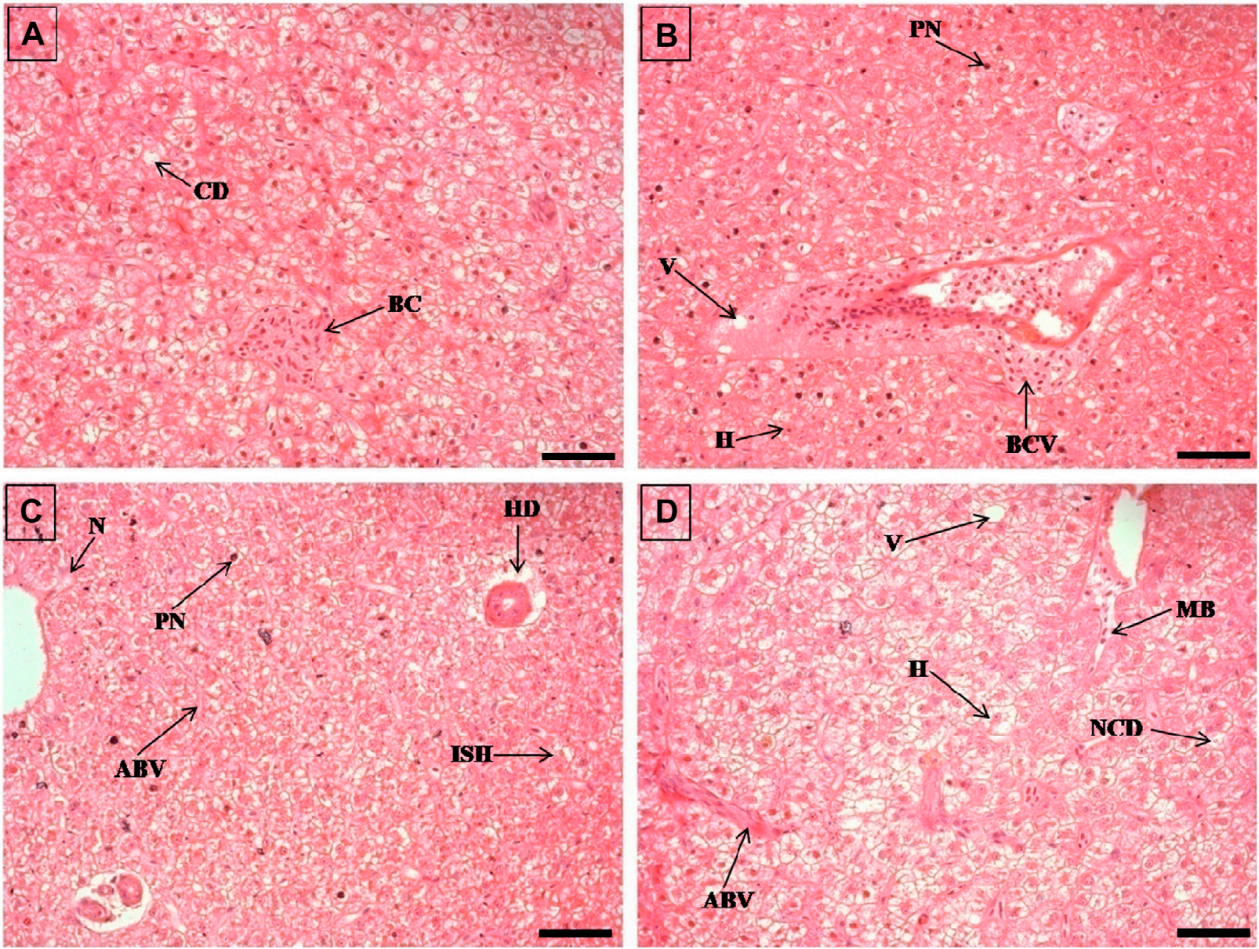
The histological sections (4 µm) of liver tissues from EB-administered golden mahseer on 21st day **(A–D)** of EB-medication. The histological changes in liver tissues of fishes fed with EB doses **(A)** at 50 μg/kg fish/day showing blood congestion (BC), cytoplsamic degeneration (CD); **(B)** at 100 μg/kg fish/day showing vacuolization (V), pyknotic nuclei (PN), hypertrophy of hepatocyte (H), blood congestion near vein (BCV); **(C)** at 250 μg/kg fish/day showing pyknotic nuclei (PN), necrosis (N), irregularly shaped hepatocytes (ISH), accumulation of blood vessels (ABV), hepatocyte degeneration (HD); **(D)** at 500 μg/kg fish/day showing vacuolization (V), hypertrophy (H), nuclear and cytoplasmic degeneration (NCD), migration of blood cells towards hepatocytes (MB), accumulation of blood vessels (ABV). The H&E stained sections are visualized at magnification of ×400 under inverted light microscope (Olympus IX53, Canada). The scale bar value depicted in the right bottom corner **(A–D)** is 20 µm.

**FIGURE 5:**
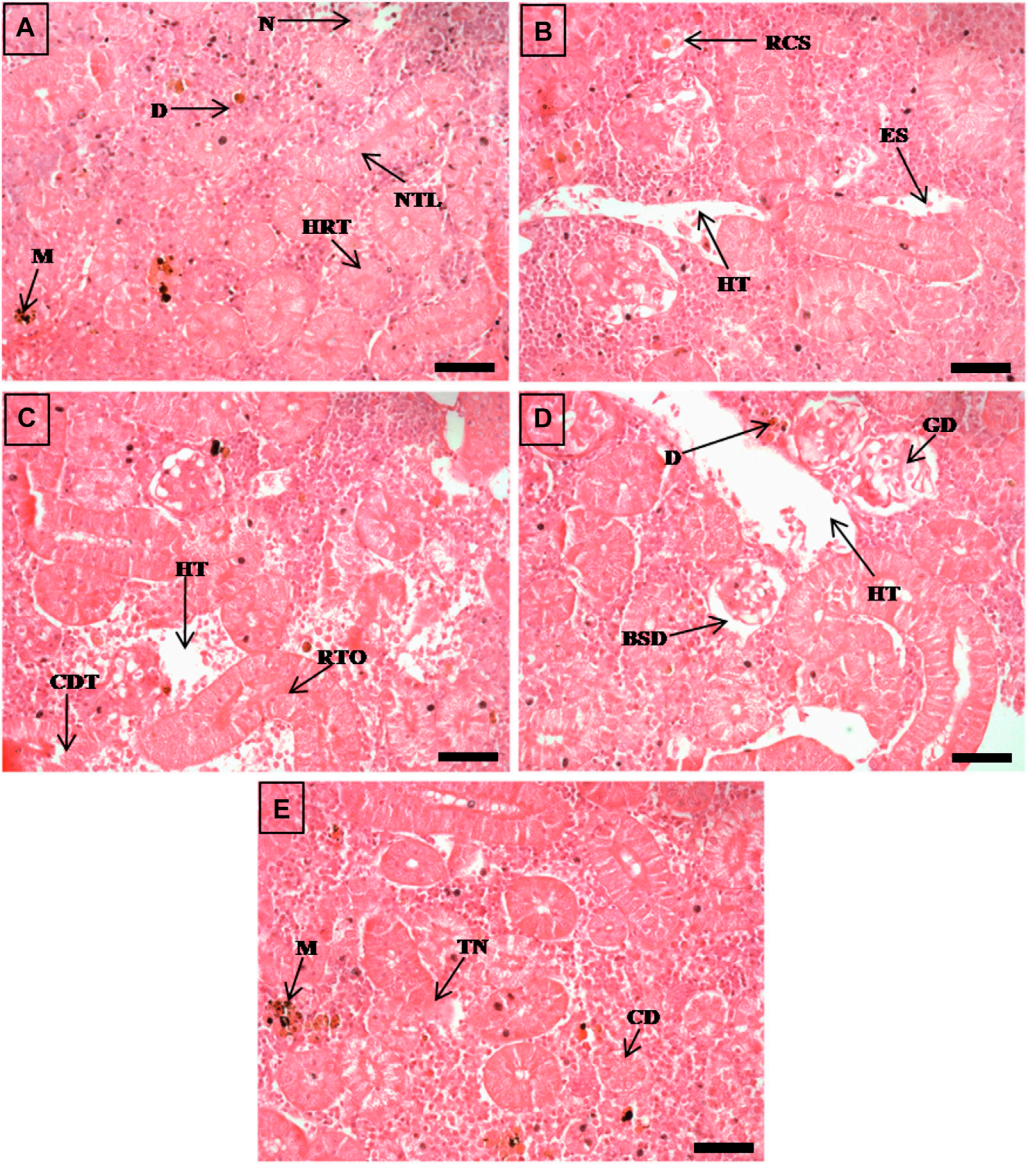
The histological sections (4 µm) of kidney tissues from EB-administered golden mahseer on 21st day **(A–E)** of EB-medication. The histological changes in kidney tissues of fishes fed with EB doses **(A)** at 50 μg/kg fish/day showing melanomacrophages (M), necrosis (N), narrowing of tubular lumen (NTL), hypertrophy in renal tubule (HRT), droplet like aggregation **(D)**; **(B)** at 100 μg/kg fish/day showing renal corpuscle shrinkage (RCS), enlarged sinusoids (ES), decreased hematopoietic tissue (HT); **(C)** at 250 μg/kg fish/day showing cellular degeneration in renal tubule (CDT), renal tubule occlusion (RTO), decreased hematopoietic tissue (HT); **(D, E)** at 500 μg/kg fish/day showing Bowman’s space dilation (BSD), glomerular degeneration (GD), decreased hematopoietic tissue (HT), droplet like aggregation **(D)**, melanomacrophages (M), tubular necrosis (TN), cloudy degeneration of tubules (CD). The H&E stained sections are visualized at magnification of ×400 under inverted light microscope (Olympus IX53, Canada). The scale bar value depicted in the right bottom corner **(A–E)** is 20 µm.

**FIGURE 6:**
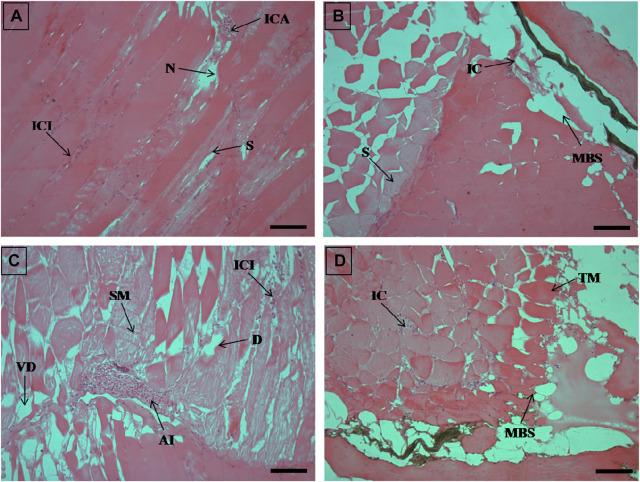
The histological sections (4 µm) of muscle tissues from EB-administered golden mahseer on 21st day **(A–D)** of EB-medication. The histological changes in muscle tissues of fishes fed with EB doses **(A)** at 50 μg/kg fish/day showing focal area of necrosis (N), splitting of myofibrils (S), inflammatory cells aggregation (ICA), infiltration of inflammatory cells between the muscle fibres (ICI); **(B)** at 100 μg/kg fish/day showing muscular bundles separation (MBS), inflammatory cells (IC), splitting of myofibrils (S); **(C)** at 250 μg/kg fish/day showing degenerative changes in muscle bundles **(D)**, severe myofibril splitting (SM), inflammatory cells infiltration (ICI), vacuolar degeneration in muscle bundles (VD), acute inflammation (AI); **(D)** at 500 μg/kg fish/day showing muscular bundles separation (MBS), marked thickening of muscle bundles (TM), inflammatory cells between the muscle fibres (IC). The H&E stained sections are visualized at magnification of ×200 under inverted light microscope (Olympus IX53, Canada). The scale bar value depicted in the right bottom corner **(A–D)** is 40 µm.

The histopathological examinations showed that prolonged higher doses of EB induces severe degenerative changes in intestine, liver, kidney and muscle tissues of golden mahseer (Table 4). The histopathological alterations can be observed on the 11th day of EB-medication in intestine (Figures 1A,B), liver (Figures 2B,C) and muscle tissues (Figures 3A–C) of golden mahseer fed with higher EB doses. The histopathological changes in intestine (Figures 1C–F), liver (Figures 4A–D), kidney (Figures 5A–E) and muscle tissues (Figures 6A–D) were observed in all the treatment groups *viz.*, 1×, 2×, 5×, and 10× EB after 21st day of medication period. The distinguishable histopathological changes in EB-fed golden mahseer included vacuolated and dilated lamina propria, necrotic debries in lumen, abundant goblet cells, disarrangement of mucosa, oedema and epithelial cell disintegration in intestine; large melanomacrophage centers, blood congestion, hypertrophy, nuclear pyknosis, vacuolization, accumulation of blood vessels, necrosis and irregularly shaped hepatocytes in liver; renal tubule degeneration and occlusion, glomerular degeneration, necrosis, dilated Bowman’s capsule and melanomacrophages in kidney and degeneration of muscle bundles, severe splitting of myofibrils, muscular oedema, melanomachrophages, increased intermuscular space, thickening of muscle fibers and inflammatory cells infiltration in muscle.

**TABLE 4:** Recovery percentage (R %) of EB metabolite Emamectin B_1a_.

Days	Concentration (µg kg^-1^)			
	50	100	250	500
0	0.60	0.60	0.60	0.60
11	161.52	943.82	1216.29	6348.31
21	196.07	429.78	1573.03	3904.49
31	134.55	351.12	217.98	2575.84
41	108.15	274.72	1101.12	1477.53
51	39.61	13.93	192.70	1174.16

### Residue depletion from muscle tissues of EB fed golden mahseer

**FIGURE 7:**
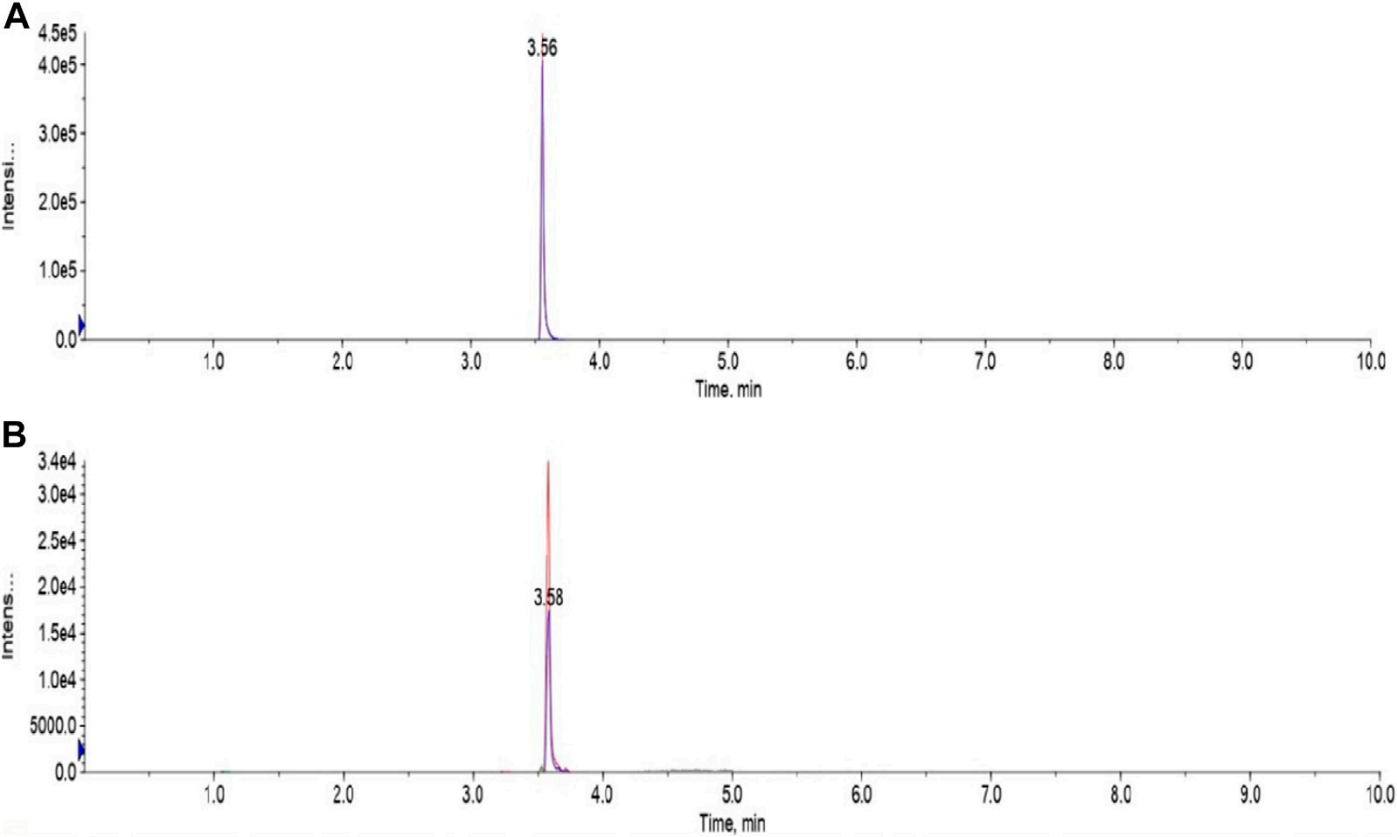
LC-MS/MS Chromatogram of Emamectin B_1a_ standard **(A)** and B_1b_ standard **(B)**.

**FIGURE 8:**
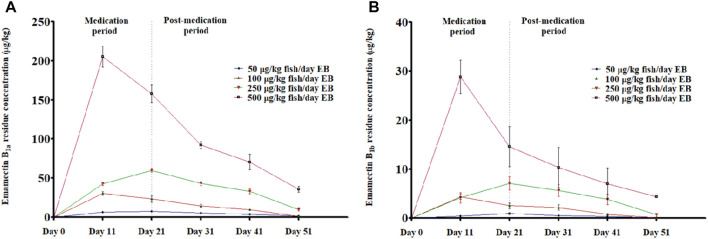
**(A, B)**: LC-MS/MS based residual analysis of EB metabolites **(A)** Emamectin B_1a_ and **(B)** Emamectin B_1b_ from muscle tissues of golden mahseer fed with different doses of EB, *viz.*, 1×: 50 μg/kg fish/day; 2×: 100 μg/kg fish/day; 5×: 250 μg/kg fish/day and 10×: 500 μg/kg fish/day for 21 days (*p* < 0.05). The error bar shows the standard error mean values.

Figure 7 showed LC-MS/MS Chromatogram of Emamectin B_1a_ standard (A) and B_1b_ standard (B). The LC-MS/MS data indicated the presence of traces of EB in the muscle of the golden mahseer fed with graded levels of EB. The results illustrate the deposition of EB in the muscle tissues within the 21 days of medication period (Figures 8A,B). The increased concentration of EB in muscle tissues were observed in the fish treated with higher concentration. 30-day post-medication period, demonstrated gradual and significant decrease in the muscle residue of EB (Figures 8A,B) (*p* < 0.05). At the end of 30 days medication period, the detected muscle Emamectin B_1a_ concentrations in 1×, 2×, 5×, and 10× treatment groups were 1.1 ± 0.8, 1.5 ± 1.0, 9.7 ± 3.3, and 37.4 ± 8.2 µg.kg^-1^, respectively (Figure 8A). The Emamectin B_1b_ residue concentrations in 1×, 2×, 5×, and 10× treatment groups were found to be 0.09 ± 0.05, 0.06 ± 0.02, 0.64 ± 0.16, and 4.34 ± 0.35 µg kg^-1^, respectively (Figure 8B).

### Determination of R %, limits of detection and limits of quantification

Tables 4–7 showed validated R %, LOD and LOQ values. EB concentrations in all samples were recorded to be greater than the LOD and LOQ values.

**TABLE 5:** Recovery percentage (R %) of EB metabolite Emamectin B_1b_.

Days	Concentration (µg kg^-1^)			
	50	100	250	500
0	66.10	66.10	66.10	66.10
11	104.56	28.49	27.86	94.30
21	47.29	2.93	27.32	62.11
31	30.20	28.35	95.44	60.68
41	40.46	59.83	0.94	20.28
51	21.88	39.32	8.35	9.54

**TABLE 6:** Validation parameters of EB metabolite Emamectin B_1a_.

Dose (µg kg^-1^)	Concentration (µg kg^-1^)		
	LOD	LOQ	R^2^
50	−32916.5	−99746.9	1.45E-05
100	−402.102	−1218.49	0.088414
250	−2618.48	−7934.8	0.002282
500	−550.799	−1669.09	0.04915

**TABLE 7:** Validation parameters of EB metabolite Emamectin B_1b_.

Dose (µg kg^-1^)	Concentration (µg kg^-1^)		
	LOD	LOQ	R^2^
50	−106.667	−323.233	0.579529
100	−2721.75	−8247.74	0.002112
250	−250.744	−759.83	0.199631
500	−77.4782	−234.782	0.723176

## Discussion

Prophylactic use of antibiotics is rare in aquaculture, while the metaphylactic use includes the practice of treating an entire population of fish orally *in toto* even if only a small percentage of the animals are affected. It is considered as a common practice in aquatic farms. In commercially produced fish species, drugs are often added to the feed to reduce handling stress, and to protect the fish health. The current model of integrated feed therapy is based on the assumption that the distribution of medicated feed is uniform to ensure that all the fish are receiving the appropriate dose of the drug (Whyte et al., 2011). The application of EB has always been preferred over the other chemicals and drugs as an antiparasitic drug. It is reported that hydrogen peroxide (H_2_O_2_), the only chemical used for the treatment of sea lice infestation, found poor in its efficacy against sea lice juvenile in British Colombia (Riccardo et al., 2007) as compared to EB (Lam et al., 2020). Among other veterinary drugs, though dichlorvos, azamethiphos, pyrethrum, cypermethrin, deltamethrin, doramectin, diflubenzuron and diflubenzuron were in application to treat crustacean ectoparasites infestation in marine fish (Horsberg, 2012; Hannisdal et al., 2020), the EB was registered as an antiparasitic veterinary drug for temperate water aquaculture in the United Kingdom in 2000 (Singha et al., 2022) best on its efficacy over other chemicals and veterinary drugs.

The present study is the first report on EB toxicity and residue depletion from muscle in golden mahseer juveniles fed with graded doses of EB *viz.*, 1× (50 μg/kg fish/day), 2× (100 μg/kg fish/day), 5× (250 μg/kg fish/day) and 10× (500 μg/kg fish/day) for 21 consecutive days. In this study, the EB was administered through medicated feed and the toxicity was assessed by survivability, feeding, behavioral characteristics and histopathological changes in the intestine, liver, kidney and muscle tissues of golden mahseer.

The *ad libitum* feeding was followed to ensure the maximum feed uptake by the golden mahseer juveniles and avoiding leaching into water. In an EB medicated trial on *L. rohita*, fishes appeared to have consumed the feed within 15–30 min of receiving the medicated diet which indicates low or undetectable amount of EB leaching into the water (Choudhary et al., 2022). However, it was observed that the feed intake by golden mahseer was not influenced by 1× and 2× EB treatments, but there was a considerable decrease in the 5× and 10× EB treated fish, even after the medication period was over. The EB medication has no impact on the palatability of the feed since there was no difference in feed consumption between the non-medicated control group and fish medicated at recommended dose as reported in Salmon (Armstrong et al., 2000) and Asian seabass (Raja et al., 2020). The nile tilapia fed with EB diet at the prescribed dose of 50 μg/kg fish/day for 7 consecutive days, appeared to consume the majority of the available feed despite an apparent trend indicating a decrease in feeding with increasing EB dose (Julinta et al., 2020). Another study on juvenile nile tilapia found that, daily feed consumption by fish ranged between 70% and 100% based on visual observation on EB treatment days (Neto et al., 2019). The feed consumption by rainbow trout was studied at two different temperatures during EB medication at dose of 50 μg/kg fish/day for 7 consecutive days, where the total feed intake was 96.4% and 99.9% by fish maintained at 6ºC and 15ºC, respectively (Roy et al., 2006). The unconsumed feed within 1 h was leftover by 5× and 10× EB treated fish, therefore rated accordingly for their feeding behavior. The decrease in the feed intake by fish is a sign of stress and intoxication (Bowker et al., 2013). The similar kind of findings were also previously reported in nile tilapia, where EB treatment at dose of 500 μg/kg fish/day showed significant decrease in feed uptake (Julinta et al., 2020). A biosafety study in Asian seabass reported 50% decrease in the feed uptake after 16th day of medication period during EB treatment at dose of 500 μg/kg fish/day for 21 consecutive days (Raja et al., 2020). Even a slight appetite decrease in fish treated with 5× and 10× EB doses did not affect the growth of the golden mahseer juveniles. This finding is supported by similar studies, where the growth of fish were not affected when administered with higher EB doses through medicated feed (Julinta et al., 2020; Raja et al., 2020; Choudhary et al., 2022). Also, the toxicity study in Atlantic salmon and rainbow trout corroborate our finding, where the growth of EB treated fish were not affected as compared to control one fed with EB-free diet (Roy et al., 2000; Bowker et al., 2013). There was no mortality recorded in golden mahseer juveniles fed with 1×, 2×, 5×, and 10× doses of EB for consecutive 21 days. The fish were able to tolerate the 10× dose of EB during and after the medication period, which is supported by EB tolerance study in Asian seabass (Raja et al., 2020) and rainbow trout (Siavash et al., 2014), where no adverse effect was observed on the survivability of EB treated fish. In contrary to above findings, 4.43% mortality was observed in nile tilapia fed with 10× EB-diet for 7 consecutive days (Julinta et al., 2020), that might be due to the toxicity imparted by EB. Various factors may be involved in determining the toxicity of a drug such as treatment duration, age, sex of the organism, species, concentration of drug, water quality, metabolism, excretion and formulation type of drug (Kumar et al., 2018; Bharti and Rasool, 2021). In the present study, no external body surface lesions were observed in control and EB-fed juveniles of golden mahseer. The behavioral characteristics of control, 1× and 2× treatment group fish were normal throughout the experimental period. The fishes of 5× and 10× treatment group showed change in behavioral characteristics as compared to control group such as lethargy, gasping of air and crowding near the inlet of water. The fish treated with 10× EB dose started showing abnormal pigmentation on their body surface at 12th day of medication period. All abnormal behavior by 5× and 10× treatment group fish were reverted back to normal stage after 9–10 days of post-medication period. Similarly, abnormal behavior was reported in Atlantic salmon, rainbow trout, nile tilapia and Asian seabass after administration of high doses of EB through medicated-feed (Roy et al., 2000; Stone et al., 2002; Julinta et al., 2020; Raja et al., 2020). Thus, treating golden mahseer juveniles with the recommended EB treatment regimen of 50 μg/kg fish/day for 7 consecutive days carried a significant safety margin of the drug.

Histopathological studies have long been recognized as reliable biomarkers of stress in fish for several reasons (van der Oost et al., 2003). In present study, the histopathological changes were observed in intestine, liver, kidney and muscle tissues of the golden mahseer juveniles fed with EB-medicated diet for 21 consecutive days. The histopathological changes were distinct at 11th and 21st day of EB medication (Figures 1–6). The observed severe degenerative changes in the organs of EB fed fish included disarrangement of mucosa, abundant goblet cells and dilated lamina propria in intestine (Figure 1); pyknotic nuclei, melanomacrophage centre, vacuolation and necrosis in liver (Figures 2, 3); degenerated renal tubules and Bowman’s capsule dilation in kidney (Figure 4) and necrosis, myofibril disintegration, muscle oedema, migration of inflammatory cells and splitting of muscle fibres in muscle (Figures 5, 6). Similar histological findings were also observed in nile tilapia fed with 1× and 10× doses of EB for 7 consecutive days, which included degenerated renal tubules in kidney, vacuolation in liver, inflammation, epithelial layer disintegration and loss of absorptive vacuoles in intestine (Julinta et al., 2020).

The MRL and acceptable daily intake (ADI) of EB is listed in the Codex Alimentarius, an international food standards published jointly by Food and Agriculture Organization (FAO) and World Health Organization (WHO) (Codex Alimentarius, 2021 (Codex)) and the records are maintained by Codex Alimentarius Commission (CAC). A meeting held by Codex Committee for pesticide residues (CCPR) suggested a MRL of 100 μg/kg fish in fillet/muscle tissues of trout and salmon for EB, which was evaluated by Joint Expert Committee on Food Additives (JECFA) from FAO/WHO. The recommended ADI of 0–0.5 μg/kg bw for a person was established by Joint FAO/WHO Expert Meeting on Pesticide Residues (JMPR), which was based on an overall No-Observed-Adverse-Effect Level (NOAEL) of 0.25 mg/kg bw per day for neurotoxicity from 14- and 53-week studies in dogs. This is supported by an NOAEL of 0.25 mg/kg bw per day from 1- and 2-year studies in rats. The MRL of 100 μg/kg fish for Emamectin B_1a_ in edible tissues of salmonid also supports the MRL established by European Union (EMEA, 2003). The Emamectin B_1a_ is considered as prominent marker for EB residue analysis from muscle tissues of the organisms in which this drug is administered (EMEA, 2003; EFSA, 2012; Codex Alimentarius, 2021). Despite the slightly reduced feeding response in 5× and 10× EB treated golden mahseer juveniles, the higher concentration of Emamectin B_1a_ was detected in muscle during medication and post medication period. The traces of Emamectin B_1a_ in muscle peaked between 11th and 21st day of medication period followed by gradual depletion in post-medication period. The results indicated that a decrease in total Emamectin B_1a_ residue concentration level was observed when EB-medicated diet was discontinued followed by feeding with EB-free diet. The muscle residue concentration of Emamectin B_1a_ was within the MRL of 100 μg/kg fish at the end of the 30 days post-medication. Here, we concluded that EB residue in muscle is within the MRL, even if it is administered for three times the recommended duration of 7 consecutive days at dose of 50 μg/kg fish/day. Similar research has been conducted in different fishes from different geographical locations of the world, where adequate margin of safety of EB was reported at recommended dose (Kim-Kang et al., 2004; Whyte et al., 2011; Collymore et al., 2014; Neto et al., 2019; Julinta et al., 2020; Raja et al., 2020; Choudhary et al., 2022). In our study, the traces of Emamectin B_1a_ were still detected in muscle tissues of EB treated golden mahseer after 30 days post-medication, which suggests the complete withdrawal of EB require more than 30 days in golden mahseer. However, the detected range falls under the MRL established by European Union and Codex Alimentarius. In Indian tropical climate, the EB withdrawal from muscle tissue of Asian seabass and nile tilapia requires 28 days and 45 days, respectively (Julinta et al., 2020; Raja et al., 2020). The withdrawal period is different in different fish species and climatic regions. A temperature dependent EB withdrawal was reported in rainbow trout (Roy et al., 2006) where, mean Emamectin B_1a_ residue concentration of 13.7 ± 10.5 ng g^−1^ at 77 days post-treatment was detected from the fillet in coldwater study. But in case of warm water study the mean Emamectin B_1a_ residue concentration was 1.6 ± 1.6 ng g^−1^ at 49 days post-treatment indicating the slow residue depletion in cold water environment. LOD is an important test that indicates presence or absence of the drug, whereas LOQ measures presence of low level of the drug. In the study, the sample concentrations of EB were observed higher as compared to LOD and LOQ values. The negative values of LOD and LOQ state negligible presence of the drug in the samples due to variability of the process, demonstrating no bias in the method.

## Conclusion

This study is the first report of the toxicity and residue depletion of EB on golden mahseer in Indian coldwater region. The feed administered EB graded levels up to 500 μg/kg fish/day for consecutive 21 days was not sufficient to cause any mortality in golden mahseer, but considerable change in feeding and behavioral characteristics were observed. The fish treated with 50 μg/kg fish/day of EB for consecutive 21 days showed no behavioral change and the muscle residue of EB was within the MRL (100 μg/kg fish) as per the guidelines of Codex Alimentarius and European Union even during medication period. This ensures the safety of recommended EB dose of 50 μg/kg fish/day for consecutive 7 days for usage as an anti-parasitic agent in aquaculture. The higher EB doses cause cellular changes in various organs such as intestine, liver, kidney and muscle of fish treated for longer duration than the recommended period, which may interfere with the detoxification and metabolism of the drug. More detailed investigation is required to elucidate the molecular mechanism by which prolonged higher EB doses lead to immunogenic and metabolic abnormalities. The consumption of meats from fish treated by recommended nominal dose of EB is safe for a person as the recommended ADI for EB is 0**–**0.5 μg/kg bw of person as established by JMPR. The study supports the safety of EB in golden mahseer and cold climate aquaculture of India at approved daily dose (ADD_farm fish_) of 50 μg/kg fish/day for 7 consecutive days to control parasitic infections. As the EB residue for nominal dose is within the MRL, no withdrawal period recommendation is required in case of golden mahseer.

## Data Availability

The original contributions presented in the study are included in the article/Supplementary Material, further inquiries can be directed to the corresponding authors.

## Abbreviations

ADD, Approved Daily Dose; ADI, acceptable daily intake; ANOVA, Analysis of Variance; CAC, Codex Alimentarius Commission; CCPR, Codex Committee for Pesticide Residues; EB, Emamectin Benzoate; EFSA, European Food Safety Authority; EMA, EMEC European Medicines Agency European Medicines Evaluation Committee; FAO, Food and Agriculture Organization; GABA, Gamma-aminobutyric Acid; IACUC, Institutional Animal Care and Use Committee; INAD, Investigational New Animal Drugs; JECFA, Joint Expert Committee on Food Additives; JMPR, Joint FAO/WHO Expert Meeting on Pesticide Residues; LC-MS/MS, Liquid Chromatography with Tandem Mass Spectrometry; LOD, Limits of Detection; LOQ, Limits of Quantification; MRL, Maximum Residue Limit; NOAEL, No-Observed-Adverse-Effect-Level; R%, Recovery Percentage; SEM, Standard Error Means; SPSS, Statistical Package for Social Science; USFDA, United States Food and Drug Administration; WHO, World Health Organization.
