# Supplementary material for: Biosafety, histological alterations and residue depletion of feed administered anti-parasitic drug emamectin benzoate in golden mahseer, Tor putitora (Hamilton, 1822) as a model candidate fish for sport fishery and conservation in temperate waters
Source: Front Pharmacol. 2023 Feb 10;14:1106124. doi: 10.3389/fphar.2023.1106124 (PMC9950520; doi:10.3389/fphar.2023.1106124)
Supplement: Supplementary file 1 [file Table1.DOCX]

**Supplementary data: Appendix A.**

Multiple reactions monitoring (MRM) transitions with source and compound parameters used for Emamectin benzoate analysis

| Name | Q1 Mass | Q3 Mass | DP  (eV) | EP  (eV) | CE  (eV) | CXP  (eV) |
| --- | --- | --- | --- | --- | --- | --- |
| Emamectin B1a_1 | 887.00 | 158.20 | 71 | 10 | 42 | 12 |
| Emamectin B1a_2 |  | 126.10 |  |  | 70 | 14 |
| Emamectin B1a_3 |  | 82.00 |  |  | 125 | 10 |
| Emamectin B1b_1 | 872.49 | 158.30 | 100 | 10 | 53 | 14 |
| Emamectin B1b_2 |  | 82.30 |  |  | 111 | 6 |
| Emamectin B1b_3 |  | 126.30 |  |  | 63 | 12 |

**Supplementary data:Appendix B.**

Experimental schedule for studying emamectin benzoate toxicity and withdrawal period in golden mahseer.

| SL No. | Parameters | Frequency |
| --- | --- | --- |
| 1. | Water quality : pH, DO, salinity, temperature, hardness, ammonia, nitrite, nitrate, oxidative reductive potential and conductivity | Twice a week |
| 2. | Animal behavior | Daily |
| 3. | Feeding behavior | Daily |
| 4. | Survival % | Once in 10 days till 30 days post-medication |
| 5. | Average body weight (g) | Once in 10 days till 30 days post-medication |
| 6. | Gross lesion: external or internal | Daily till 10 days post-medication |
| 7. | Histopathological examination: Intestine, Liver, Kidney and Muscle | After 11^th^ and 21^st^ day of medication period |
| 8. | Collection of muscle tissues for EB residue analysis | Day 0, 11 and 21 (medication-period) and once in every 10 days after the medication period till 30 days post-medication. |
