# Supplementary figures and images for: Biosafety, histological alterations and residue depletion of feed administered anti-parasitic drug emamectin benzoate in golden mahseer, Tor putitora (Hamilton, 1822) as a model candidate fish for sport fishery and conservation in temperate waters
Source: Front Pharmacol. 2023 Feb 10;14:1106124. doi: 10.3389/fphar.2023.1106124 (PMC9950520; doi:10.3389/fphar.2023.1106124)

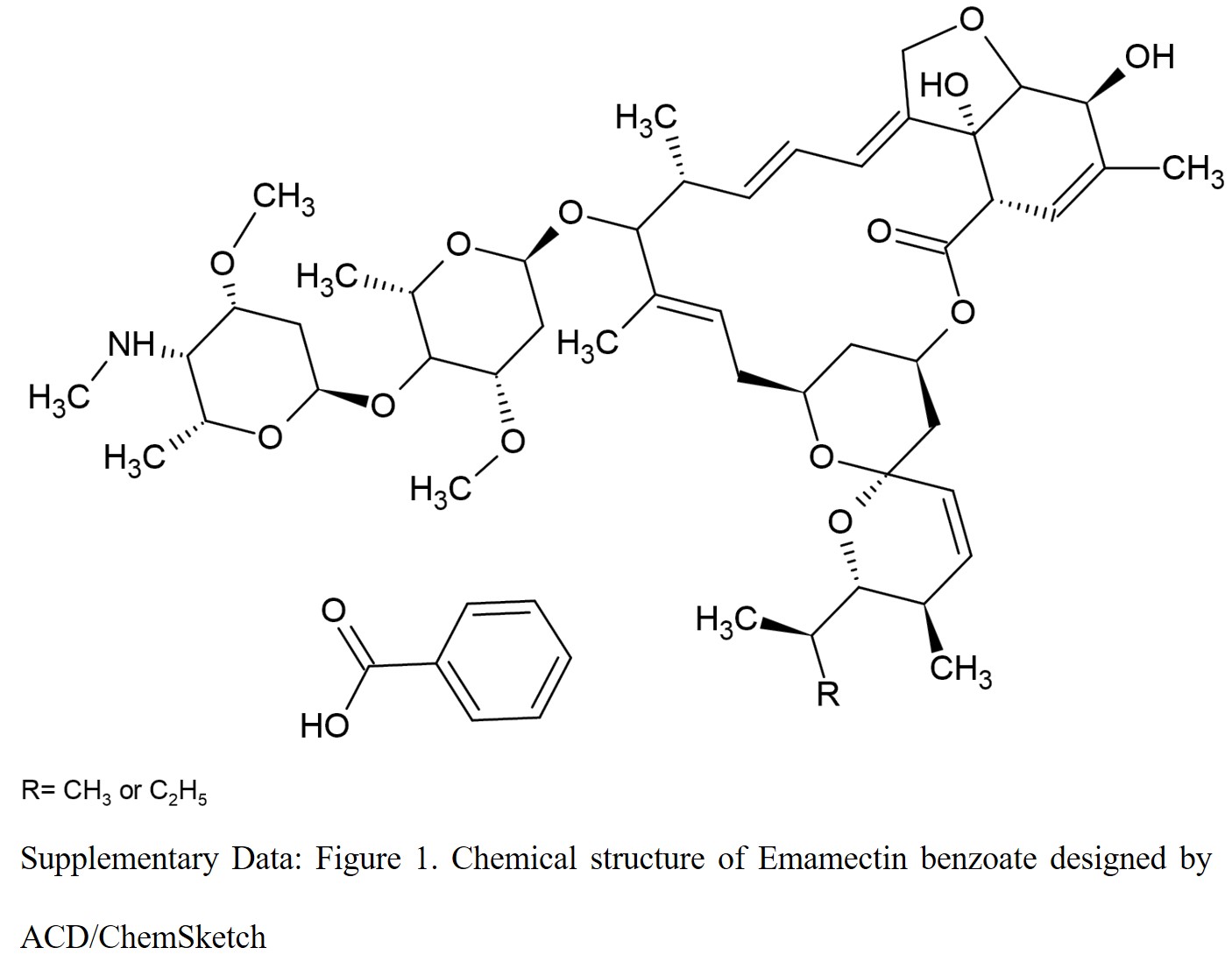

Supplement: Supplementary file 2 [file Image2.JPEG]

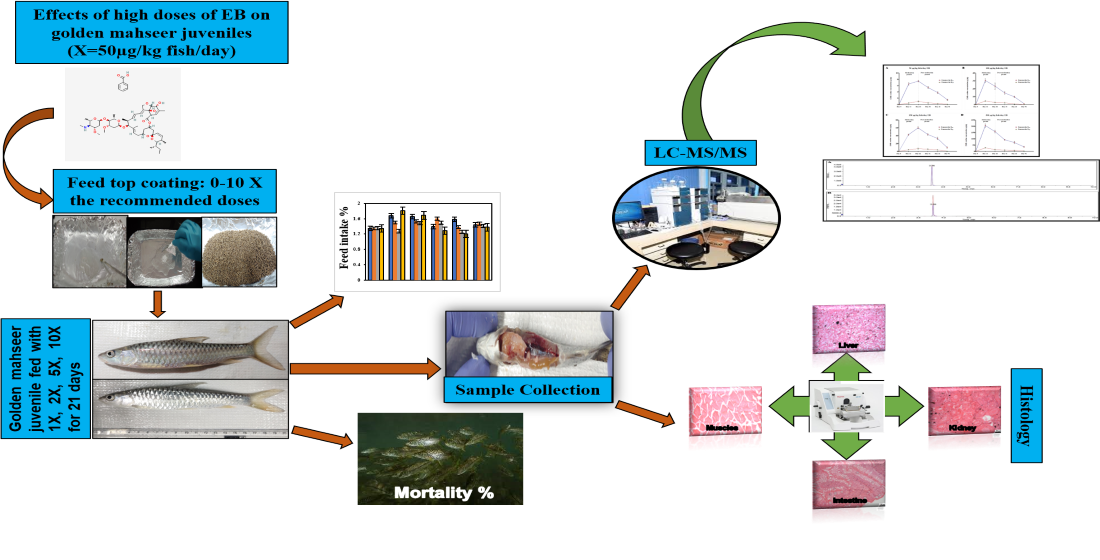

Supplement: Supplementary file 3 [file Image1.PNG]
